# Large Intestinal Obstruction and Perforation From Metastatic Merkel Cell Carcinoma: A Case Report

**DOI:** 10.7759/cureus.44467

**Published:** 2023-08-31

**Authors:** David Lee, Melanie Roman, Garrett L Newman, Yamil Lopez, Zane W Ashman, Michael P O'Leary

**Affiliations:** 1 Surgery, Loma Linda University Medical Center, Loma Linda, USA; 2 Surgery, Loma Linda University Health, Murrieta, USA; 3 Pathology, Loma Linda University Health, Murrieta, USA

**Keywords:** peritoneal carcinomatosis, metastatic disease, bowel perforation, bowel obstruction, merkel cell carcinoma

## Abstract

Merkel cell carcinoma (MCC) is a rare and aggressive neuroendocrine neoplasm of the skin that has a high propensity to metastasize. Abdominal metastases of MCC have been described previously though these are typically regional with nodal spread. We report the case of a 60-year-old man with a history of left upper extremity MCC who had resection, radiation therapy, and immunotherapy. He ultimately developed large bowel obstruction from metastatic intraperitoneal implants. A 6 cm mass at the descending colon was biopsied and proven to be metastatic MCC. The tumor eroded through the wall of the colon and perforated, requiring emergent colectomy for septic shock. Herein, we describe the first case of colonic perforation secondary to metastatic MCC. This case illustrates the importance of expedient and multifactorial management of patients with rapidly growing metastatic colonic tumors that are at risk for perforation.

## Introduction

Merkel cell carcinoma (MCC) is a rare and aggressive neuroendocrine neoplasm of the skin that commonly presents in the head and neck region as a painless red/purple nodule [1]. The pathogenesis of this tumor remains yet to be elucidated, but several studies have established a causal relationship between Merkel cell polyomavirus (MCPyV) infection and subsequent MCC in 80% of cases [1,2]. Additional risk factors precipitating the development of MCC include UV exposure, advancing age, and immunosuppression, which likely aids MCPyV host genome integration [1]. Because of its aggressive nature, about one-third of patients diagnosed with MCC have distant metastatic disease at diagnosis. Common sites of metastatic disease include lymph nodes, skin/body wall, and liver [3,4]. We present a rare case of upper extremity MCC, which metastasized to the intraperitoneal cavity-causing obstruction and ultimate perforation. This report reviews the relevant literature and discusses the management of this atypical metastatic pattern.

## Case presentation

A 60-year-old male, with a history of left upper extremity MCC and clinically palpable axillary lymph nodes, underwent curative intent resection three years prior. After this, he developed mediastinal metastases for which he received radiation and systemic immunotherapy. Within a year, he presented with diffuse metastatic disease, including peritoneal carcinomatosis. Given the unusual finding of peritoneal disease, he underwent a percutaneous biopsy of the peritoneal nodularity, which was consistent with his primary MCC. In the acute setting, he presented to the hospital with constipation and obstipation. Workup, including computed tomography (CT) of the abdomen and pelvis again, showed carcinomatosis with dilated loops of small and large bowel with a transition point at a mass in the descending colon. The patient was admitted, and conservative measures attempted to allow for appropriate resuscitation, including nasogastric tube decompression, intravenous fluids, and NPO. After stabilization, he underwent laparoscopic diverting transverse colostomy. The patient had a return of bowel function and relief of the distention and was subsequently discharged postoperative day two. He then presented postoperative day four with acute onset abdominal pain, hypotension, and leukocytosis. He progressed into septic shock requiring intubation and vasopressor support. 

**Figure 1:**
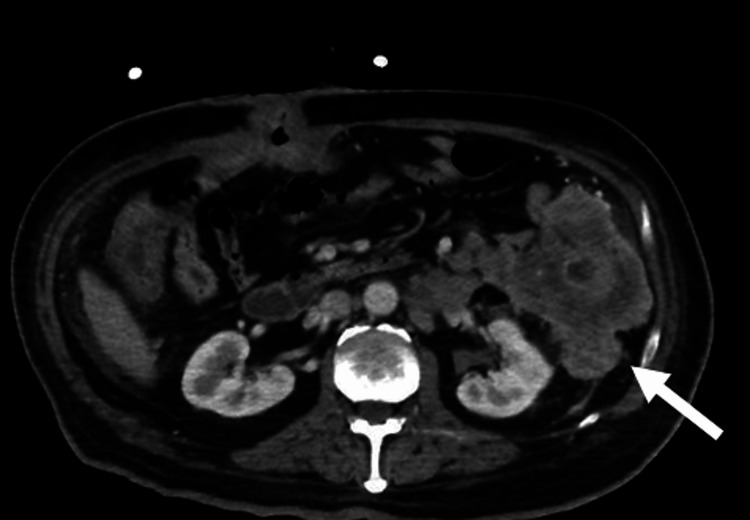
Preoperative CT abdomen and pelvis showing the outpouching of the posterior descending colon, which is consistent with the tumor invading through the colonic wall.

A CT abdomen and pelvis showed an intact colostomy, but retroperitoneal perforation of bowel contents at the site of the descending colon mass with abscess formation (Figure 1). The patient's complete blood count showed a white blood cell count of 11,550 per microliter (normal range: 4,500-11,000 per microliter), with 85.4% neutrophil predominance and a decreased hemoglobin of 10.4 g/dL (normal range: 13.8-17.2 g/dL). A comprehensive metabolic panel showed findings including hyponatremia of 128 mmol/L (normal range: 136-145 mmol/L).

Given the patient's deteriorating condition, the decision was made to proceed to the operating room for palliative intent resection. Intraoperatively, the omentum was densely adherent to the left lower quadrant requiring omentectomy. After extensive dissection, the left colic branch was identified, and the pedicle was transected. The extended left colectomy was completed, and source control was accomplished. Postoperatively, the patient was weaned off vasopressors but remained intubated in the ICU. He was extubated on postoperative day one and was ultimately transitioned to an oral diet. The patient elected for comfort care and was discharged home on home hospice.

**Figure 2:**
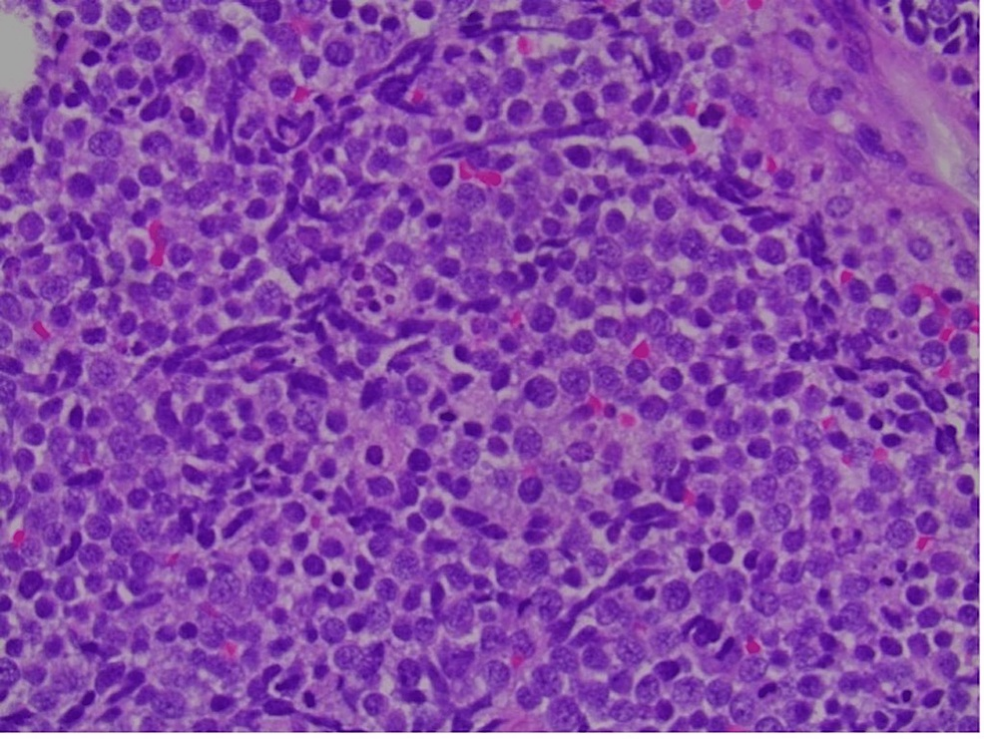
400X magnification of tumor cells showing small round blue cell tumor cells with a high N:C ratio and finely dispersed chromatin (salt and pepper). Nuclear molding and crush artifact are also evident.

The resected colonic specimen showed metastatic MCC that extended into the peri-colonic and abdominal wall soft tissue. Necrosis with acute inflammatory exudate was appreciated. In the resected omentum, metastatic MCC was present as well. The tumor also showed positive staining for CK20, cytokeratin cocktail, and synaptophysin, markers consistent with MCC (Figure 2). The patient was staged at stage IV, pT4N3M1c [5].

## Discussion

The incidence of MCC in the United States ranges from 0.66 to 0.79 cases per 100,000 people. The incidence has been rising over time in most areas by a factor of 3-5 from 1985 to 2013, which could be attributed to an aging population and increased detection of cancer [6]. MCC is an aggressive neuroendocrine carcinoma of the skin that has a high rate of local recurrence, lymph node metastases, and distant metastases [7]. MCC commonly presents in older patients with fair skin as a rapidly growing red-purple nodule. Diagnosis of primary MCC is confirmed by histopathology. Imaging is indicated whenever metastatic or unresectable disease is suspected based on history and physical findings such as enlarged or tender lymphadenopathy [1].

In the case of local MCC, management of the primary tumor is stratified based on whether the patient has at least one of these following risk factors: primary tumor larger than 1 cm, chronic T cell immunosuppression, HIV, CLL, solid organ transplant, head/neck primary site, or lymphovascular invasion. If the patient does not have any of these risk factors, then excision with 1-2 cm margins is appropriate, and re-excision or adjuvant radiotherapy can be administered if there are positive margins. If a patient does have any of the following risk factors, then a multidisciplinary assessment is done to determine the margins of excision, and adjuvant radiotherapy will always follow. Along with excision of the primary tumor site, a sentinel lymph node biopsy is also recommended, and if suspected to be positive, nodal dissection, adjuvant chemotherapy, or radiation therapy can be considered. Management of regional MCC is akin to local MCC with the distinction being in the management of the draining nodal basin when there is metastatic disease [2,8].

In the case of our patient who presented with stage IV disease, guidelines suggest that, after a multidisciplinary consultation and comprehensive imaging, systemic therapy within a clinical trial is preferred if available. If not, a combination of systemic therapy, radiotherapy, and resection of limited metastasis can be considered. The preferred systemic interventions include PD-L1 inhibitors. Immunotherapy has been shown to provide similar response rates to those of chemotherapy and may provide greater durability of response [9]. If patients have adverse immune-related events with checkpoint immunotherapies, then a variety of chemotherapy regimens can be considered, such as cisplatin with or without etoposide [6]. Further work must be done to determine the optimal utilization of each treatment modality for patients with MCC who present with diffuse metastatic disease.

Abdominal metastatic disease is described, but rare. Girard et al. [3] identified 27 out of 111 patients with abdominal metastases from MCC to the non-regional lymph nodes, liver, and pancreas. A study that analyzed a cohort of over 200 patients found that the primary site of initial MCC influences the location of metastasis [10]. Gastrointestinal metastases from MCC are particularly rare, and the stomach has been reported as the most common site [3,11]. MCC metastasis causing large bowel obstruction is atypical, and its clinical presentation must be elucidated further to guide future management.

We report a rare case of stage IV MCC causing large bowel obstruction and an ultimate large bowel perforation. There have been several proposed mechanisms that explain the aggressiveness of this neoplasm. One study elucidated a signaling pathway involving class 1 phosphoinositide 3 kinase (PI3K) and Akt kinase. MCC uses Akt kinase to phosphorylate downstream targets, such as Mdm2 to induce p53 protein degradation and promote constant cell cycle maturation. The antagonist of PI3K that dephosphorylates PIP3, PTEN, has also been shown to be deleted in MCC, which allows it to readily transduce signal cascades leading to cell growth [12]. Another study analyzed the immunohistochemical profiles of more aggressive MCC and found statistically significant correlations between metastatic tumor spread and overexpression of matrix metalloproteinase, VEGF, and NF-kappaB [13]. MCC has been shown to have a high capacity for penetrating vascular walls and attaining lymphovascular invasion even in the early stages of pathogenesis [14].

## Conclusions

This case highlights the importance of recognizing the early metastatic spread of MCC. While the intraperitoneal spread of this tumor histology is rare, it should remain on the differential. The rapid progression of this patient’s disease highlights the need for a multidisciplinary approach to the care of these patients. These groups should include medical oncology, surgical oncology, radiation oncology, palliative care, and social workers.

In summary, this is the first case reported in the scientific literature of colonic perforation due to carcinomatosis from MCC metastasis. Intestinal involvement from MCC can be rapidly progressive and potentially fatal. In turn, this should prompt earlier intervention and discussion of goals of care.
